# Antibacterial Activity of Commercial Phytochemicals against *Aeromonas* Species Isolated from Fish

**DOI:** 10.3390/pathogens8030142

**Published:** 2019-09-08

**Authors:** Barbara Kot, Kinga Kwiatek, Justyna Janiuk, Małgorzata Witeska, Agnieszka Pękala-Safińska

**Affiliations:** 1 Department of Microbiology, Faculty of Natural Sciences, Siedlce University of Natural Sciences and Humanities, 14 Bolesława Prusa Str., 08-110 Siedlce, Poland; 2 Department of Animal Physiology, Faculty of Natural Sciences, Siedlce University of Natural Sciences and Humanities, 14 Bolesława Prusa Str., 08-110 Siedlce, Poland; malgorzata.witeska@uph.edu.pl; 3 Department of Fish Diseases, National Veterinary Research Institute, 57 Partyzantów Ave., 24-100 Puławy, Poland; A.Pekala@piwet.pulawy.pl

**Keywords:** phenolic acid, essential oils, trans-cinnamaldehyde, *Aeromonas hydrophila*, *Aeromonas salmonicida* subsp. *salmonicida*, *Aeromonas sobria*

## Abstract

Antimicrobial activities of phytochemicals—*trans*-cinnamaldehyde (TC), ferulic acid (FA), *p*-coumaric acid (*p*-CA), caffeic acid (CA), chlorogenic acid (CHA), *Thymus vulgaris* essential oil (TO), *Eugenia caryophyllus* essential oil (ECO), and *Melaleuca alternifolia* oil (TTO) against *Aeromonas* species—were assessed. Growth of all *Aeromonas salmonicida* subsp. *salmonicida* and almost all *Aeromonas sobria* strains was inhibited by TC at concentration 0.01 mg/mL, and for most *Aeromonas hydrophila* strains minimal inhibitory concentrations (MIC) ranged from 0.01 to 0.19 mg/mL. The inhibitory effect of TC against *A. salmonicida* subsp. *salmonicida* was comparable to the effect of oxytetracycline, and in the case of *A. salmonicida* subsp. *salmonicida* and *A. sobria* was higher compared to gentamicin. MIC of FA, *p*-CA, and CA for most strains ranged from 1.56 to 3.12 mg/mL, and MIC values of TO for most strains ranged from 0.39 to 0.78 mg/mL. TO and TC at the concentrations below ½ MIC values used in mixtures exhibited strong synergism. ECO and TC showed synergy in mixture of ⅛ MIC of ECO and ¼ MIC of TC. TC and TO exhibited the strongest inhibitory and bactericidal effect against investigated *Aeromonas* species, and they are a promising alternative to the use of antibiotics in controlling the growth of these fish pathogens.

## 1. Introduction

Species belonging to the genus *Aeromonas* are ubiquitous and cosmopolitan gram-negative opportunistic bacteria commonly present in fresh water and often cause ulcer disease or hemorrhagic septicemia in fish [1,2]. Different species of *Aeromonas* cause severe skin lesions and anemia in fish resulting in high mortality [3,4]. According to Rasmussen-Ivey et al. [5], virulence in *Aeromonas hydrophila* is multifactorial, including production and/or secretion of adhesins, cytotoxins, hemolysins, lipases, and proteases accompanied by the ability of biofilm formation. It is particularly dangerous under intensive aquaculture conditions since stress caused by high stocking density and fishery management may suppress fish immune mechanisms and increase a risk of disease outbreak and accelerate disease transmission. Outbreaks of *A. hydrophila* infections are related to increased susceptibility of fish caused by adverse environmental conditions, such as hypoxia or nitrogenous waste buildup [5]. Experimental infection of healthy perch with *A. sobria* which was isolated from an affected farm fish resulted in the development of clinical signs such as focal skin lesion and fin rot, which indicates that this species is involved in the mortality of the farmed perch and it has the potential to act as a primary pathogen of perch [6]. *A. salmonicida*, responsible for furunculosis in salmonids, also can infect many warm and cold water, freshwater and marine species of fish [7].

The use of antibiotics in aquaculture is restricted due to their possible adverse effects on environment, and health of consumers [8]. Antibiotics may accumulate in fish tissues, suppress immune response and promote selection of antibiotic-resistant bacteria [9]. Gbylik-Sikorska et al. [10] measured concentrations of veterinary antibiotics (aminoglycosides, β-lactams, diaminopyrimidines, fluoroquinolones, lincosamides, macrolides, pleuromutilins, sulfonamides, and tetracyclines) in 159 fresh water, 443 fish, and 150 sediment samples from Polish rivers and lakes. They reported that the levels of antibiotics ranged from 5 μg/kg to 125 μg/kg in fish samples, from 0.02 μg/L to 10 μg/L in water samples, and from 1 μg/kg to 8 μg/kg in sediment samples. Aminoglycosides, macrolides and β-lactams showed the highest concentrations. These data indicate that antibiotics are commonly present in aquatic environment and in fish, and thus they may pose a serious risk of disturbance in aquatic microorganism communities and a danger of development of antibiotic-resistant bacteria. Antibiotics present in the environment may also directly affect health of fish and their consumers. In this situation, there is a need to find alternative therapies to control bacterial diseases in fish, to reduce the emergence of bacterial resistance to antibiotics and to avoid public health concerns.

Therefore, herbal treatments seem to be reasonable alternatives to antibiotic use in fish and many studies proved that plant extracts or isolated herbal active compounds are effective against *A. hydrophila* by both enhancing nonspecific immune response of fish and/or direct antibacterial activity [11,12,13,14]. Herbal products seem to be a promising source of bioactive compounds, being available and inexpensive [9].

The aim of this study was to evaluate the antimicrobial activity of selected secondary plant metabolites against pathogenic *Aeromonas* species isolated from fish.

## 2. Results

### 2.1. Antimicrobial Activity of Phytochemicals against Aeromonas Species

**Figure 1:**
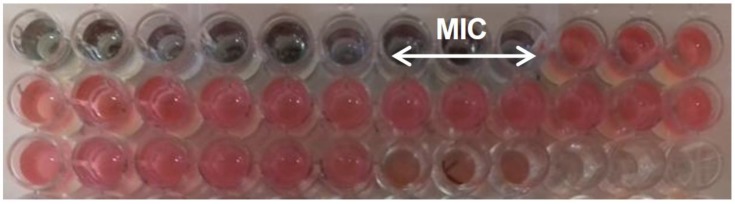
Determination of minimal inhibitory concentrations (MIC) of phytochemicals by using the resazurin microtiter-plate assay. The lowest concentration of the phytochemicals at which no resazurin color change from blue to pink occurred, confirmed inhibition of metabolic activity of bacterial cells by phytochemicals, and was taken as the MIC value. Each test was repeated three times.

Antibacterial activity of different phytochemicals against three *Aeromonas* species pathogenic for fish were evaluated in vitro by measurement of minimal inhibitory concentrations (MIC) (Figure 1) and minimum bactericidal concentrations (MBC) values.

*Trans*-cinnamaldehyde (TC) showed the highest antibacterial activity with the lowest MIC values ranging from 0.01 mg/mL to 0.19 mg/mL in case of the most *A. hydrophila* strains. For two *A. hydrophila* strains isolated from kidney and necrotic changes in common carp gills, the MIC values were 0.39 and 0.78 mg/mL, respectively. Meanwhile, the growth of *A. salmonicida* subsp. *salmonicida* and almost all *A. sobria* strains were inhibited at concentration 0.01 mg/mL of TC (Table 1).

The MIC values of TC for growth of *A. hydrophila* and *A. sobria* were higher than the MIC values of oxytetracycline (from 0.002 to 0.01 mg/mL) but in the case two *A. hydrophila* strains (Pt552, Pt572) the MIC values of antibiotic (0.39 mg/mL) were higher compared to TC. Meanwhile, the sensitivity of *A. salmonicida* subsp. *salmonicida* to TC and oxytetracycline was the same ( Table 1; Table 2). Comparison of the MIC values of TC and gentamicin showed that almost all *A. hydrophila* strains were more sensitive to gentamicin than TC but three strains had the same degree of sensitivity to both agents, whereas, the MIC values of TC inhibiting growth of *A. salmonicida* subsp. *salmonicida* and *A. sobria* were smaller (0.01 mg/mL) compared to MIC values of gentamicin (0.05 and 0.09 mg/mL). The MBC values of TC ranged from 0.01 mg/mL to 0.78 mg/mL. The same value of MBC as MIC was found in for six strains, while MBC equal to 2 MIC, 4 MIC, or 8 MIC were obtained for five, four, and one strain, respectively (Table 1).

Phenolic acids such as ferulic acid (FA), *p*-coumaric acid (*p*-CA), caffeic acid (CA) also effectively inhibited growth of *Aeromonas* species, although the MIC values were higher than those of TC and for most strains they were in the range of 1.56–3.12 mg/mL. Sensitivity of investigated *Aeromonas* species to chlorogenic acid (CHA) was lower than to other phenolic acids because the MIC values for the most strains ranged from 3.12 mg/mL to 6.25 mg/mL (Table 1). The MBC values of FA, *p*-CA and CA were equal to MIC, 2MIC, or 4 MIC for the most of strains and ranged from 1.56 mg/mL to 6.24 mg/mL. The MBC values of CHA for most strains amounted 12.5 mg/mL and the MBC to MIC ratio was 4.

Among tested essential oils (EOs), *Thymus vulgaris* essential oil (TO) had the highest antibacterial activity against *Aeromonas* species. In case of the most strains, the MIC values of TO ranged from 0.39 to 0.78 mg/mL. The MBC values of TO were the same as value of MIC in the case of nine *Aeromonas* strains and ranged from 0.09 mg/mL to 0.78 mg/mL. The sensitivity of investigated species to *Eugenia caryophyllus* essential oil (ECO) was more variable, because the MIC values for the most *A. hydrophila* strains ranged from 0.38 mg/mL to 1.56 mg/mL. Sensitivity of *A. salmonicida* subsp. *salmonicida* and *A. sobria* to ECO was higher compared to *A. hydrophila* because MIC values were in range 0.01 to 0.38 mg/mL. The MBC to MIC ratio in case of half of strains was 2, while for the remaining strains was equal to 1, 4, or 8. The investigated *Aeromonas* species showed lower sensitivity to *Melaleuca alternifolia* oil (TTO) than to other tested EOs. The MIC values of TTO in case of most strains were in the range 0.78–3.12 mg/mL and growth of three *A. hydrophila* strains was inhibited at concentration 12.5 mg/mL (Table 2). The MBC to MIC ratio in the case of most strains was 4 and MBC values ranged from 1.56 mg/mL to 50 mg/mL.

### 2.2. Antimicrobial Interaction of Chosen Phytochemicals

The phytochemicals (TC, TO, ECO, FA and *p*-CA) that showed high antibacterial activity when used alone, were also used to determine their antibacterial activity in mixtures against *A. hydrophila* strains for which the obtained MIC values were highest. The MIC values of mixtures containing TO and TC, ECO and TC, and FA, and *p*-CA were presented in Table 3. The obtained results showed that phytochemicals in mixtures inhibited growth of these strains at lower concentrations than used alone. Mixtures of TO and TC at the concentrations below MIC exhibited strong synergism (fractional inhibitory concentration indices (FICI) values were 0.30 and 0.36) or demonstrated additive antibacterial effect (FICI < 1). Between TO and TC no interactions occurred when TC in mixtures was at MIC concentration (FICI values > 1). ECO and TC also showed synergy when their mixture contained ⅛ MIC of ECO and ¼ MIC of TC (FICI = 0.37). FA and *p*-CA demonstrated an additive antibacterial effect (FICI = 0.75). Between phytochemicals in mixtures no interactions occurred when the compounds were used at concentration equal to MIC.

## 3. Discussion

Plant products showing antibacterial activity are interesting and promising compounds to control disease problems in aquaculture production. Many plants produce phytochemicals with different mechanisms of action that are active against a wide range of pathogenic microorganisms [15]. Their antimicrobial activity is related to chemical diversity and structural complexity. The use of antibiotics to prevent fish diseases results in economic loss for companies in the aquaculture industry and above all pollutes the environment and causes the development of antibiotic-resistant bacteria [16]. Plants had been used since ancient times for their medicinal properties. High antimicrobial potential of phytochemicals and lack of any apparent emergence of bacterial resistance to them make plant products valuable subjects of research.

The present study demonstrates the antibacterial activity of selected phytochemicals and indicates that they are a promising alternative to the use of antibiotics in controlling the growth of fish pathogens such as *A. hydrophila*, *A. salmonicida* subsp. *salmonicida* and *A. sobria*. Among tested plant metabolites TC had highest activity against investigated *Aeromonas* species. The growth of *A. salmonicida* subsp. *salmonicida* and of almost all *A. sobria* strains was inhibited at concentration 0.01 mg/mL, and in case of most *A. hydrophila* strains, MIC ranged from 0.01 mg/mL to 0.19 mg/mL. Results obtained by Abdelhamed et al. [17] showed that TC had better antibacterial activity against *A. hydrophila* ML09-119 strain than other tested plant-derived compounds and the MIC value was 80 µl/mL. In our study, we investigated activity of TC against 10 *A. hydrophila* strains and the MIC values varied but in case of five strains they were close to or lower than those obtained by Abdelhamed et al. [17]. TC is a phenolic compound extracted from bark of cinnamon that shows low toxicity and is recognized and classified as safe for addition to foods by the U.S. Food and Drug Administration [18]. The research conducted by Abdelhamed et al. [17] showed that all intestinal fragments from catfish fed TC-supplemented diets at 10, 20, 30, and 40 mg/kg were healthy with no signs of ulcer, necrotic enterocytes, and inflammation. Moreover, these studies showed that the addition of TC to the catfish diet increased the number of goblet cells, which are responsible for synthesis and secretion of mucins that form a mucus layer on the intestine surface. Mucous layer protects the host against microbial pathogens, prevents gastrointestinal pathology, and participates in nutrient digestion and absorption [19]. In our preliminary study assessing toxicity of orally administered TC to common carp no significant hematological alterations were found in fish (unpublished data). The data reported by Bickers et al. [20] suggest that cinnamaldehyde is safe to rats when administered by oral route as a single dose (2220 mg/kg) and to mice when administered repeatedly for even over 2 years (up to 550 mg/kg/day). Cinnamaldehyde is oxidized to benzoic acid derivatives, which are conjugated with glycine or glucuronic acid and excreted primarily in the urine [21]. Cinnamaldehyde excretion rate at 24 h after administration varies between 70 and 98% in rodents, depending on the route of administration; and reaches 100% within 8 h when given orally to healthy human volunteers [20,22]. The cytotoxicity of cinnamaldehyde was also determined by Ribeiro et al. [23] by using a fibroblast cell line, and the results showed that cinnamaldehyde did not compromise fibroblast cell viability. It was observed that action of TC results in damage of cell wall and membrane of treated bacteria, and consequently to loss of inner cell material, cell lysis and leakage of cellular contents [17,24]. TC exhibits significant antimicrobial properties against various pathogens, including uropathogenic *Escherichia coli* [25], methicillin-resistant *Staphylococcus aureus* [26] and other Gram-positive and Gram-negative bacteria [27]. In our study, we showed that higher concentrations of TC (0.19–0.78mg/mL) were necessary to inhibit growth of some *A. hydrophila* strains but other fish pathogens such as *A. sobria* and *A. salmonicida* subsp. *salmonicida* were inhibited at low concentrations of TC. Yilmaz et al. [16] also showed that *trans*-cinnamic acid had strong inhibitory effect on *A. sobria* and exhibited moderate inhibition of *A. salmonicida*. In our research, growth of these fish pathogens was inhibited at lower concentrations of TC than in research of Yilmaz et al. [16]. Different sensitivity of strains may be due to the differences in the cell wall composition and structure thereby reduce the cell wall penetration by TC or *trans*-cinnamic acid. The differences may also result from different methods and different culture media used during determination of MIC. In our research, the determination of the MIC values of the tested agents were carried out by the resazurin microtiter-plate assay which allowed to determine metabolic activity of bacterial cells. This assay is effective for reliable assessment of antibacterial activity of phytochemicals against bacteria, which was confirmed in our earlier studies [28] and by other authors [29]. Our research indicated that TC can be very effective for treatment of infection in fish caused by *A. sobria* and *A. salmonicida* subsp. *salmonicida*. The inhibitory effect of TC against *A. salmonicida* subsp. *salmonicida* is comparable with to inhibitory effect of oxytetracycline because the same MIC values were obtained for these two agents. Meanwhile, the inhibition of *A. salmonicida* subsp. *salmonicida* and *A. sobria* growth required the use of higher concentrations of gentamicin than TC. 

Hydroxycinnamic acids such as FA, *p*-CA, and CA also effectively inhibited growth of *Aeromonas* species, although the MIC values were higher than those for the TC. Meanwhile, the sensitivity of investigated *Aeromonas* species to CHA was smaller than to other phenolic acids. According to our knowledge, it is first study concerning the effects of these compounds against *Aeromonas* spp. The antibacterial activity of FA is due to its membrane-targeting action, which causes loss of membrane integrity [30]. Borges et al. [31] showed that MBC value of FA for *E. coli* was 2500 μg/mL and 5000 μg/mL for *S. aureus*. Our results showed that MBC values for majority of *A. hydrophila* strains were higher (3120–6240 μg/mL) and for *A. salmonicida* subsp. *salmonicida* and *A. sobria* 6240 μg/mL. According to Lou et al. [32], *p*-CA has a dual mechanism of bactericidal activity, because it increased the outer and plasma membrane permeability and bound to genomic DNA, which might have affected replication, transcription, and expression. *p*-CA mechanism of action may be associated with damage of cell membrane and reduction of respiratory activity [32]. Vaquero et al. [33] also showed that CA as a phenolic acid shows relatively strong nucleophilic properties. Kępa et al. [34] investigated the antibacterial activity of the CA and observed diverse effects on *S. aureus* strains with MICs varying from 256 μg/mL to 1024 μg/mL. In our study, the MIC values for *Aeromonas* species in the most cases were higher and ranged from 390 μg/mL to 12,500 μg/mL. The results obtained by Lou et al. [35] showed that CHO effectively inhibited the growth of tested bacterial pathogens (*Staphylococcus aureus*, *Streptococcus pneumoniae*, *Bacillus subtilis*, *E. coli*, *Shigella dysenteriae*, *Salmonella* Typhimurium), and the MIC values ranged from 20 to 80 µg/mL. Our studies indicated that CHO was less effective against *Aeromonas* species, and higher concentrations should be used to inhibit the growth of these microorganisms. In our research, among tested essential oils, TO showed the highest antibacterial activity against *Aeromonas* species, although the MIC values of TO were higher than TC. Quendera et al. [36] also investigated antimicrobial activity of commercial TO against *Aeromonas* species and obtained MIC values were between 0.47 and 1.9 mg/mL. In our study, the MIC values of TO for most strains ranged from 0.39 to 0.78 mg/mL. Differences in the MIC values of TO are due to the different composition of the oils used. The most important compounds of TO with antimicrobial properties are thymol (68.1%) and carvacrol (3.5%) from phenol group, and the monoterpene hydrocarbons such as *p*-cymene (11.2%) and γ-terpinene (4.8%) [37]. Contents of the phenolic compounds such as thymol and carvaclor significantly affect antibacterial properties of EOs that have the greatest bactericidal activities, followed by aldehydes, ketones, alcohols, ethers and hydrocarbons [38]. Thymol and carvacrol are isomers with similar chemical structures and likely to have similar mechanisms of antimicrobial activity [39]. The antibacterial properties of these compounds are associated with their lipophilic character and their accumulation in cell membranes. These compounds can increase membrane fluidity and permeability, by disturbance of membrane embedded proteins causing alteration of ion transport processes and inhibition of respiration [40]. The other EOs investigated in our study also inhibited growth of bacteria but higher concentrations were required. Our results were similar to those obtained by Starliper et al. [41] who found out that clove oil and TTO, respectively, moderately or minimally inhibited growth of *A. salmonicida* subsp. s*almonicida*. The main component of clove oil is eugenol, which reaches from 30 to 95%, whereas eugenol acetate content amounts up to 22% [42]. The major constituents of commercial TTO are terpinen-4-ol, γ-terpinene, 1,8-cineole, α-terpinene, α-terpineol, *p-*cymene, and α-pinene [43].

According to the classification by Soro et al. [44], when the MBC to MIC ratio is greater than 4, a phytochemical is considered bacteriostatic, whereas it is bactericidal when this ratio is lower or equal to 4. In our research, in the case of most *Aeromonas* strains this ratio showed bactericidal effect of investigated phytochemicals. However, for some *A. hydrophila* strains MIC values of tested phytochemicals were higher. For this reason, we checked antimicrobial effects of some tested phytochemicals in combinations to improve their antibacterial properties. According to our knowledge, very limited information is available on the mechanism of combined action of phytochemicals. Our study showed that synergism between TO and TC was observed occurred when the ratios TO/TC in the mixture were ¼ MIC/⅛ MIC, ⅛ MIC/¼ MIC or 1/16 MIC/¼ MIC, respectively, while combinations with ½ MIC were additive. The synergistic effect of TO and *Cinnamomum zeylanicum* oil against *S. aureus* was found by Kon and Rai [45]. The combination of pair of components showing synergistic effects reduced the concentration needed to yield the same microbial effect when compared with the sum of the purified components [46]. In our research, strong synergistic effect of TO and TC against *A. hydrophila* K894 strain was observed and concentrations of both agents were reduced from over 80% to about 87%, depending on the ratio of components. Pei et al. [47] showed that synergistic effect of TO and TC against *E. coli* resulted in effective reduction of concentration by 25%. Synergistic effects of TO and TC is probably related to an increase in permeability of the cytoplasmic membrane under the influence of thymol or carvacrol that enables cinnamaldehyde more easily enter the cell. Other hypothesis assumes that thymol or carvacrol could increase the number or size of the pores created by the binding of TC to proteins in the cell membrane [46]. In our study, we also obtained synergistic effect ECO and TC against *A. hydrophila* K848 strain. Main constituents of ECO are eugenol and eugenol acetate TC can pass through the phospholipid membrane of bacteria and bind to proteins. The alteration of membrane permeability and the loss of functional proteins transporting molecules leads to cytoplasmic coagulation, denaturation of several enzymes and proteins, and the loss of metabolites and ions [48]. The synergistic effect of eugenol and TC is probable due to the interaction of these components with different proteins [47].

## 4. Materials and Methods

### 4.1. Bacterial Strains

Sixteen strains belonging to *Aeromonas* genus were obtained from the National Veterinary Research Institute in Puławy (Table 4). The strains were obtained from different species of fish with or without clinical symptoms of infection caused by these bacteria. Biochemical identification system API 20NE (BioMerieux, Marcy-I’Étoile, Lyon, France) was used for identification of the *Aeromonas* species. *Aeromonas salmonicida* subsp. *salmonicida* used in this study were mesophilic strains.

### 4.2. Phytochemicals

The following phytochemicals were used in the study: *trans*-cinnamaldehyde (TC) (lot no. MKBW8907V), FA (lot no. BCBM6076V), *p*-coumaric acid (*p*-CA) (lot no. BCBV5232), CA (lot no. 100M1247V), chlorogenic acid (CHA) (lot no. SLBV3345) (Sigma-Aldrich, Steinheim, Germany) (Figure S1), oils of *Thymus vulgaris* (thyme essential oil, TO) (lot no. 951562), *Eugenia caryophyllata* (eugenia caryophyllus essential oil, ECO) (lot no. 980442) and *Melaleuca alternifolia* (tee tree oil, TTO) (lot no. 10306) (ETJA, Elbląg, Poland).

### 4.3. Determination of MIC of Phytochemicals

Antimicrobial effects of phytochemicals were evaluated using a serial two-fold dilution method. Phytochemicals such as TC, FA, *p*-CA, CA and CHA were initially diluted in dimethyl sulfoxide (DMSO) (Sigma-Aldrich) (1:1 *v/v*), and then in Mueller-Hinton Broth (MHB; BBL, Becton Dickinson, Sparks, Md.). The EOs were initially diluted in 96% ethanol, and then in MHB. Serial two-fold dilutions for TC were prepared to obtain concentrations ranging from 50 mg/mL to 0.002 mg/mL, in case of CA, CHA and TO concentrations ranged from 50 mg/mL to 0.02 mg/mL, and for FA, *p*-CA and TTO from 50 mg/mL to 0.19 mg/mL. Determination of MIC of tested phytochemicals was carried out according to method described by Kot et al. [23]. Aliquots of 100 µL (two-fold dilutions) of tested phytochemicals were transferred in triplicates to wells of tissue culture polystyrene 96-well plate (Nunclon, Roskilde, Denmark). Bacterial strains were grown on the MH agar for 18 h at 28 °C. The cells of each strain were suspended in the sterile phosphate-buffered saline (PBS) to get the optical density OD_565_ = 0.3 (densitometer DEN-1, Biosan, Latvia). The obtained suspensions of the cells were diluted 1:100 (*v/v*) with MHB and transferred (100 µL) to the wells of earlier prepared microplates containing two-fold dilutions of tested phytochemicals. Final concentration of bacterial cells in the wells was approximately 5 × 10^5^ CFU/mL. Control of bacterial growth was performed in the wells with cell suspensions without phytochemicals, without phytochemicals with DMSO or ethanol (at concentrations used in the dilutions) as well as control of sterility of the media (wells without addition of cell suspension). The inoculated microplates were incubated without agitation at 28 °C for 24 h. Determination of MIC values of the tested agents was carried out by the resazurin microtiter-plate assay which allowed to determine metabolic activity of bacterial cells. For this purpose, 10 µL of sterile resazurin water solution (0.01%) (Sigma-Aldrich) was added to each well. After that, the microplates were incubated for 2 h in darkness at 28 °C. The change of color from blue to pink indicated reduction of resazurin by live bacterial cells. The lowest concentration of the phytochemicals at which no resazurin color change was observed which confirmed inhibition of metabolic activity of bacterial cells by phytochemicals, was taken as the MIC value. Each test was repeated three times. MIC of phytochemicals were compared with MIC of antibiotics used in fish: oxytetracycline hydrochloride (Ichtioxan, Biofaktor, Skierniewice, Poland) and gentamycin (Biowet, Puławy, Poland).

### 4.4. Determination of MBC

Mixtures (100 µL) from wells showing no metabolic activity of bacterial cells were subcultivated on MH agar and incubated at 28 °C for 24 h. After incubation, the MBC were defined as the lowest concentrations of phytochemicals, at which less than 5 colonies were observed on MH agar plate. Each test was repeated three times. MBC of phytochemicals were compared with MBC of antibiotics used in fish: oxytetracycline hydrochloride and gentamycin.

### 4.5. Antimicrobial Interaction of Phytochemicals

The antimicrobial effect of phytochemical combinations was investigated for one concentration above and several concentrations below the MIC of each compound in mixture (Tables S1–S5). Combinations of two different phytochemicals were prepared by adding 50 µL of each to the same well of tissue culture polystyrene 96-well plate that was subsequently inoculated with 100 µL suspension of the bacterial cells. The final concentration of the bacterial cells in the well was approximately 5 × 10^5^ CFU/mL. After incubation for 24 h at 28 °C, the MIC for each combination was determined by the resazurin microtiter-plate assay. The interaction between two phytochemicals was determined by calculating the fractional inhibitory concentration (FIC) with FIC indexes (FICI) [49]. FICI were interpreted as follows: synergy, FICI ≤ 0.5; additivity, 0.5 < FICI ≤ 1; indifference (no effect), 1 < FICI ≤ 2 and FICI > 2, antagonism [50].

## 5. Conclusions

Recently an increase in content of antibiotics in the aquatic environment and in fish is observed, and thus they may pose a serious risk of disturbance in aquatic microorganism communities and a danger of development of antibiotic-resistant bacteria. Therefore, development of the non-chemical and natural therapeutics became necessary. Our results indicate that all used phytochemicals presented inhibitory effect against three *Aeromonas* species that can be pathogenic to fish. The MIC and MBC values, and ratio MBC/MIC showed that TC and TO exhibited the strongest inhibitory and bactericidal effect against these species, which indicates the possibility of their use as environmentally friendly antibacterial agents to prevent and control *A. hydrophila*, *A. salminicida* subsp. s*almonicida* and *A. sobria* in aquaculture. TC may even be more effective than antibiotics for treatment of fish infections caused by *A. sobria* and *A. salmonicida* subsp. s*almonicida* because for growth inhibition of these pathogens higher concentrations of gentamicin were required compared to TC, and the inhibitory effect of TC against *A. salmonicida* subsp. s*almonicida* was comparable with inhibitory effect of oxytetracycline. TC and TO showed the synergistic effect against *A. hydrophila* which reduced effective MIC. Reduction of MIC value of individual components is important because allows of the use of lower concentrations of these substances as antibacterial supplements in fish feed. Future in vivo studies are required, including detailed toxicological evaluation of TC effects on healthy fish, determining of the optimum dosage of TC in fish feeds and evaluation of TC clinical usefulness to treat *Aeromonas* infection.

## Figures and Tables

**Table 1:** Antibacterial activity of *trans*-cinnamaldehyde and phenolic acids against *Aeromonas* species isolated from fish.

Strain	MIC and MBC of Phytochemicals (mg/mL)									
	*trans*-Cinnamaldehyde		Ferulic Acid		*p*-Coumaric Acid		Caffeic Acid		Chlorogenic Acid	
	MIC	MBC	MIC	MBC	MIC	MBC	MIC	MBC	MIC	MBC
*A. hydrophila*										
K822	0.09	0.36	3.12	6.24	1.56	3.12	1.56	3.12	6.25	12.5
K848	0.39	0.39	3.12	3.12	1.56	1.56	3.12	3.12	6.25	12.5
K858	0.19	0.19	1.56	1.56	1.56	3.12	1.56	3.12	6.25	12.5
K887	0.19	0.76	6.24	6.24	1.56	6.24	3.12	3.12	12.5	12.5
K894	0.78	0.78	1.56	6.24	1.56	3.12	3.12	3.12	6.25	12.5
Pt552	0.02	0.04	1.56	6.24	1.56	6.24	3.12	3.12	0.78	3.12
K865	0.09	0.36	3.12	3.12	1.56	3.12	1.56	1.56	12.5	12.5
Pt 572	0.01	0.04	3.12	6.24	1.56	6.24	0.78	3.12	3.12	3.12
Sc17	0.09	0.18	1.56	3.12	1.56	6.24	1.56	6.24	3.12	12.5
Pło3	0.19	0.38	1.56	3.12	1.56	3.12	12.5	100	3.12	12.5
*A. salmonicida* subsp. *salmonicida*										
K908	0.01	0.01	1.56	6.24	1.56	6.24	0.78	6.24	3.12	12.5
K914	0.01	0.08	1.56	6.24	1.56	6.24	1.56	3.12	3.12	12.5
*A. sobria*										
Pt 559	0.01	0.02	-	-	-	-	0.39	1.56	1.56	6.24
Sc15	0.01	0.01	1.56	6.24	1.56	6.24	1.56	3.12	3.12	12.5
Ok 1	0.19	0.38	3.12	6.24	1.56	3.12	6.25	50	3.12	12.5
Pło2	0.01	0.01	1.56	6.24	1.56	3.12	3.12	3.12	3.12	12.5

“-“ undefined.

**Table 2:** Antibacterial activity of essential oils and conventional antibiotics against *Aeromonas* species isolated from fish.

Strain	MIC and MBC (mg/mL)									
	Thyme Oil		Eugenia Caryophyllus Oil		Tee Tree Oil		Oxytetracycline		Gentamicin	
	MIC	MBC	MIC	MBC	MIC	MBC	MIC	MBC	MIC	MBC
*A. hydrophila*										
K822	0.39	0.78	0.78	1.56	0.78	1.56	0.003	0.024	0.09	0.18
K848	0.78	0.78	1.56	3.12	12.5	50	0.006	0.024	0.05	0.1
K858	0.39	0.39	1.56	3.12	12.5	12.5	0.006	0.024	0.09	0.09
K887	1.56	6.24	1.56	3.12	3.12	12.5	0.006	0.024	0.05	0.1
K894	0.78	1.56	1.56	3.12	12.5	12.5	0.006	0.024	0.09	0.18
Pt552	0.39	1.56	0.19	1.52	3.12	12.5	0.39	1.56	0.02	0.04
K865	0.39	0.39	0.78	3.12	0.78	3.12	0.006	0.024	0.05	0.1
Pt 572	0.39	0.78	0.38	1.52	1.56	6.24	0.39	0.78	0.09	0.1
Sc17	0.09	0.09	0.38	0.38	0.78	3.12	0.01	0.02	0.09	0.09
Pło3	0.78	0.78	0.78	1.56	3.12	12.5	0.03	0.024	0.05	0.2
*A. salmonicida* subsp. *salmonicida*										
K908	0.39	0.39	0.19	0.19	3.12	6.24	0.01	0.04	0.05	0.2
K914	0.78	3.12	0.05	0.4	0.78	3.12	0.01	0.01	0.05	0.05
*A. sobria*										
Pt 559	-	-	0.01	0.02	-	-	0.002	0.012	-	-
Sc15	0.39	0.39	0.19	0.38	3.12	3.12	0.006	0.024	0.09	0.36
Ok 1	0.78	0.78	0.19	0.19	0.78	3.12	0.01	0.02	0.09	0.18
Pło2	0.09	0.09	0.38	0.76	0.78	3.12	0.003	0.006	0.09	0.18

“-“ undefined.

**Table 3:** Antimicrobial activity against *A. hydrophila* of phytochemical combinations.

Strain	Ratio (mg/mL)	FIC		FICI	Effect
	TO:TC	FIC_TO_	FIC_TC_		
K894	½ MIC (0.39):⅛ MIC (0.1)	0.50	0.13	0.63	Additivity
	¼ MIC (0.19):⅛ MIC (0.1)	0.24	0.13	0.37	Synergy
	⅛ MIC (0.1):¼ MIC (0.19)	0.13	0.24	0.36	Synergy
	1/16 MIC (0.05):¼ MIC (0.19)	0.06	0.24	0.30	Synergy
	1/32 MIC (0.02):½ MIC (0.39)	0.02	0.50	0.52	Additivity
K848	MIC (0.78):1/16 MIC (0.02)	1.0	0.05	1.05	No effect
	½ MIC (0.39):½ MIC (0.19)	0.50	0.48	0.98	Additivity
	¼ MIC (0.19): ½MIC (0.19)	0.24	0.48	0.72	Additivity
	⅛ MIC (0.1): ½MIC (0.19)	0.13	0.48	0.61	Additivity
	1/16 MIC (0.05): MIC (0.39)	0.06	1.0	1.06	No effect
	1/32 MIC (0.02): MIC (0.39)	0.03	1.0	1.03	No effect
	ECO:TC	FIC_ECO_	FIC_TC_		
K848	⅛ MIC (0.2): ¼ MIC (0.1)	0.13	0.25	0.37	Synergy
	½ MIC (0.78): ⅛ MIC (0.05)	0.50	0.12	0.62	Additivity
	MIC (1.56):1/16 MIC (0.02)	1.0	0.05	1.05	No effect
	MIC (1.56):1/32 MIC (0.01)	1.0	0.02	1.02	No effect
	FA:*p*-CA	FIC_FA_	FIC*_p_*_-CA_		
K865	¼ MIC (0.78): ½ MIC (0.78)	0.25	0.50	0.75	Additivity
K887	¼ MIC (1.56): ½ MIC (0.78)	0.25	0.50	0.75	Additivity

TO—thyme essential oil, TC—*trans*-cinnamaldehyde, ECO—eugenia caryophyllus essential oil, FA—ferulic acid, *p*-CA—*p*-coumaric acid, FIC—fractional inhibitory concentration, FICI—fractional inhibitory concentration indices.

**Table 4:** *Aeromonas* species used in the study.

Species	Number of Strain	Year of Isolation	Source
*Aeromonas hydrophila*	K822	2014	*Cyprinus carpio* (skin ulcer)
	K848	2015	*Cyprinus carpio* (kidney)
	K858	2015	*Cyprinus carpio* (kidney)
	K887	2015	*Cyprinus carpio* (skin ulcer)
	K894	2015	*Cyprinus carpio* (gill necrosis)
	Pt552	2015	*Oncorhynchus mykiss* (kidney)
	K865	2015	*Cyprinus carpio* (kidney)
	Pt 572	2016	*Oncorhynchus mykiss* (dead fish)
	Sc17	2014	*Cyprinus carpio* (kidney)
	Pło3	2014	*Rutilus rutilus* (kidney)
*Aeromonas salmonicida* subsp. *salmonicida*	K908	2016	*Cyprinus carpio* (kidney)
	K914	2016	*Cyprinus carpio* (gill necrosis)
*Aeromonas sobria*	Pt 559	2016	*Oncorhynchus mykiss* (dead fish)
	Sc15	2015	*Oncorhynchus mykiss* (skin ulcer)
	Ok 1	2014	*Perca fluviatilis* (kidney)
	Pło2	2014	*Rutilus rutilus* (kidney)

## Supplementary Materials

The following are available online at https://www.mdpi.com/2076-0817/8/3/142/s1, Figure S1: Chemical structure of trans-cinnamaldehyde, ferulic, p-coumaric, caffeic and chlorogenic acids. Table S1: Concentrations used in study of antimicrobial interaction of TO and TC against K894 strain. Table S2: Concentrations used in study of antimicrobial interaction of TO and TC against K848 strain. Table S3: Concentrations used in study of antimicrobial interaction of ECO and TC against K848 strain. Table S4: Concentrations used in study of antimicrobial interaction of FA and p–CA against K865 strain. Table S5: Concentrations used in study of antimicrobial interaction of FA and p–CA against K887 strain.
