# Supplementary material for: Antibacterial Activity of Commercial Phytochemicals against Aeromonas Species Isolated from Fish
Source: Pathogens. 2019 Sep 8;8(3):142. doi: 10.3390/pathogens8030142 (PMC6789499; doi:10.3390/pathogens8030142)
Supplement: Supplementary file 1 [file pathogens-08-00142-s001.pdf]

Table S1. Concentrations used in study of antimicrobial interaction of TO and TC against K894 strain.

Concentration of TC (mg/ml)	Concentration of TO (mg/ml)						
	2 MIC (1.56)	MIC (0.78)	½ MIC (0.39)	¼ MIC (0.19)	⅛ MIC (0.1)	1/16 MIC (0.05)	1/32 MIC(0.02)
2 MIC (1.56)	1.56/1.56	0.78/1.56	0.39/1.56	0.19/1.56	0.1/1.56	0.05/1.56	0.02/1.56
MIC (0.78)	1.56/0.78	0.78/0.78	0.39/0.78	0.19/0.78	0.1/0.78	0.05/0.78	0.02/0.78
½ MIC (0.39)	1.56/0.39	0.78/0.39	0.39/0.39	0.19/0.39	0.1/0.39	0.05/0.39	0.02/0.39
¼ MIC (0.19)	1.56/0.19	0.78/0.19	0.39/0.19	0.19/0.19	0.1/0.19	0.05/0.19	0.02/0.19
⅛ MIC (0.1)	1.56/0.1	0.78/0.1	0.39/0.1	0.19/0.1	0.1/0.1	0.05/0.1	0.05/0.1
1/16 MIC (0.05)	1.56/0.05	0.78/0.05	0.39/0.05	0.19/0.05	0.1/0.05	0.05/0.05	0.02/0.05
1/32 MIC (0.02)	1.56/0.02	0.78/0.02	0.39/0.02	0.19/0.02	0.1/0.02	0.05/0.02	0.02/0.02

TO - thyme essential oil, TC - *trans*-cinnamaldehyde

Table S2. Concentrations used in study of antimicrobial interaction of TO and TC against K848 strain.

Concentration of TC (mg/ml)	Concentration of TO (mg/ml)						
	2 MIC (1.56)	MIC (0.78)	½ MIC (0.39)	¼ MIC (0.19)	⅛ MIC (0.1)	1/16 MIC (0.05)	1/32 MIC (0.02)
2 MIC (0.78)	1.56/0.78	0.78/0.78	0.39/0.78	0.19/0.78	0.1/0.78	0.05/0.78	0.02/0.78
MIC (0.39)	1.56/0.39	0.78/0.39	0.39/0.39	0.19/0.39	0.1/0.39	0.05/0.39	0.02/0.39
½ MIC (0.19)	1.56/0.19	0.78/0.19	0.39/0.19	0.19/0.19	0.1/0.19	0.05/0.19	0.02/0.19
¼ MIC (0.1)	1.56/0.1	0.78/0.1	0.39/0.1	0.19/0.1	0.1/0.1	0.05/0.1	0.02/0.1
⅛ MIC (0.05)	1.56/0.05	0.78/0.05	0.39/0.05	0.19/0.05	0.1/0.05	0.05/0.05	0.05/0.02
1/16 MIC (0.02)	1.56/0.02	0.78/0.02	0.39/0.02	0.19/0.02	0.1/0.02	0.05/0.02	0.02/0.02
1/32 MIC (0.01)	1.56/0.01	0.78/0.01	0.39/0.01	0.19/0.01	0.1/0.01	0.05/0.01	0.02/0.01

TO - thyme essential oil, TC - trans-cinnamaldehyde

Table S3. Concentrations used in study of antimicrobial interaction of ECO and TC against K848 strain.

Concentration of TC (mg/ml)	Concentration of ECO (mg/ml)						
	2 MIC (1.56)	MIC (0.78)	½ MIC (0.39)	¼ MIC (0.19)	⅛ MIC (0.1)	1/16 MIC (0.05)	1/32 MIC (0.02)
2MIC (0.78)	1.56/0.78	0.78/0.78	0.39/0.78	0.19/0.78	0.1/0.78	0.05/0.78	0.02/0.78
MIC (0.39)	1.56/0.39	0.78/0.39	0.39/0.39	0.19/0.39	0.1/0.39	0.05/0.39	0.02/0.39
½ MIC (0.19)	1.56/0.19	0.78/0.19	0.39/0.19	0.19/0.19	0.1/0.19	0.05/0.19	0.02/0.19
¼ MIC (0.1)	1.56/0.1	0.78/0.1	0.39/0.1	0.19/0.1	0.1/0.1	0.05/0.1	0.02/0.1
⅛ MIC (0.05)	1.56/0.05	0.78/0.05	0.39/0.05	0.19/0.05	0.1/0.05	0.05/0.05	0.05/0.02
1/16 MIC (0.02)	1.56/0.02	0.78/0.02	0.39/0.02	0.19/0.02	0.1/0.02	0.05/0.02	0.02/0.02
1/32 MIC (0.01)	1.56/0.01	0.78/0.01	0.39/0.01	0.19/0.01	0.1/0.01	0.05/0.01	0.02/0.01

ECO - eugenia caryophyllus essential oil, TC - trans-cinnamaldehyde

Table S4. Concentrations used in study of antimicrobial interaction of FA and *p*–CA against K865 strain.

Concentration of *p*-CA (mg/ml)	Concentration of FA (mg/ml)						
	2 MIC (6.24)	MIC (3.12)	½ MIC (1.56)	¼ MIC (0.78)	⅛ MIC (0.39)	1/16 MIC (0.19)	1/32 MIC (0.1)
2MIC (3.12)	6.24/3.12	3.12/3.12	1.56/3.12	0.78/3.12	0.39/3.12	0.19/3.12	0.1/3.12
MIC (1.56)	6.24/1.56	3.12/1.56	1.56/1.56	0.78/1.56	0.39/1.56	0.19/1.56	0.1/1.56
½ MIC (0.78)	6.24/0.78	3.12/0.78	1.56/0.78	0.78/0.78	0.39/0.78	0.19/0.78	0.1/0.78
¼ MIC (0.39)	6.24/0.39	3.12/0.39	1.56/0.39	0.78/0.39	0.39/0.39	0.19/0.39	0.1/0.39
⅛ MIC (0.19)	6.24/0.19	3.12/0.19	1.56/0.19	0.78/0.19	0.39/0.19	0.19/0.19	0.1/0.19
1/16 MIC (0.1)	6.24/0.1	3.12/0.1	1.56/0.1	0.78/0.1	0.39/0.1	0.19/0.1	0.1/0.1
1/32 MIC (0.05)	6.24/0.05	3.12/0.05	1.56/0.05	0.78/0.05	0.39/0.05	0.19/0.05	0.1/0.05

FA - ferulic acid, *p*-CA - *p*-coumaric acid

Table S5. Concentrations used in study of antimicrobial interaction of FA and *p*–CA against K887 strain.

Concentration of *p*-CA (mg/ml)	Concentration of FA (mg/ml)						
	2 MIC (12.48)	MIC (6.24)	½ MIC (3.12)	¼ MIC (1.56)	⅛ MIC (0.78)	1/16 MIC (0.39)	1/32 MIC (0.19)
2 MIC (3.12)	12.48/3.12	6.24/3.12	3.12/3.12	1.56/3.12	0.78/3.12	0.39/3.12	0.19/3.12
MIC (1.56)	12.48/1.56	6.24/1.56	3.12/1.56	1.56/1.56	0.78/1.56	0.39/1.56	0.19/1.56
½ MIC (0.78)	12.48/0.78	6.24/0.78	3.12/0.78	1.56/0.78	0.78/0.78	0.39/0.78	0.19/0.78
¼ MIC (0.39)	12.48/0.39	6.24/0.39	3.12/0.39	1.56/0.39	0.78/0.39	0.39/0.39	0.19/0.39
⅛ MIC (0.19)	12.48/0.19	6.24/0.19	3.12/0.19	1.56/0.19	0.78/0.19	0.39/0.19	0.19/0.19
1/16 MIC (0.1)	12.48/0.1	6.24/0.1	3.12/0.1	1.56/0.1	0.78/0.1	0.39/0.1	0.19/0.1
1/32 MIC (0.05)	12.48/0.05	6.24/0.05	3.12/0.05	1.56/0.05	0.78/0.05	0.39/0.05	0.19 /0.05

FA - ferulic acid, *p*-CA - *p*-coumaric acid

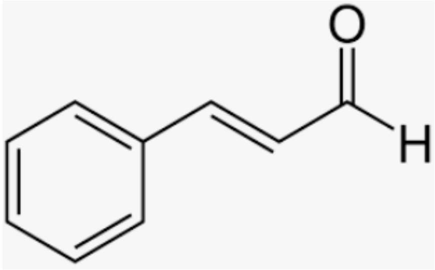

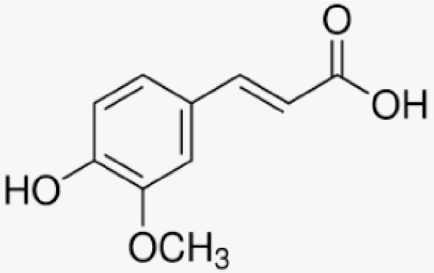

trans-**cinnamaldehyde** **ferulic acid**

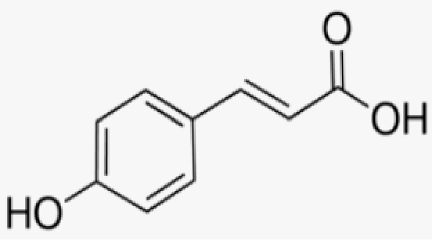

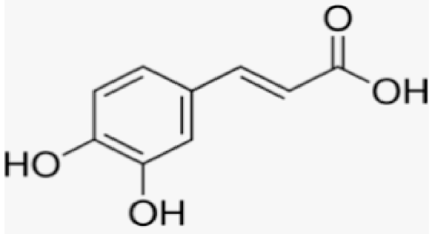

***p*-coumaric acid** **caffeic acid**

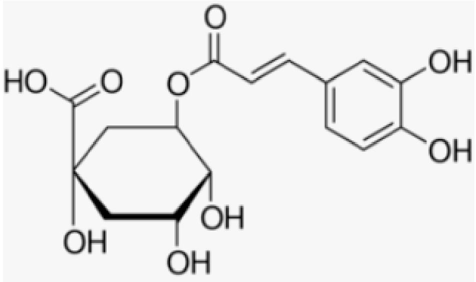

chlorogenic acid

Figure S1. Chemical structure of *trans*-cinnamaldehyde, ferulic, *p*-coumaric, caffeic and chlorogenic acids.
